# Development and Validation a Nomogram for Predicting Overall Survival in Patients With Intrahepatic Cholangiocarcinoma

**DOI:** 10.3389/fsurg.2021.659422

**Published:** 2021-05-17

**Authors:** Chen Yuan, Zhigang Hu, Kai Wang, Shubing Zou

**Affiliations:** Department of Hepatobiliary Surgery, The Second Affiliated Hospital of Nanchang University, Nanchang, China

**Keywords:** intrahepatic cholangiocarcinoma, prognosis, nomogram, overall survival, SEER

## Abstract

**Background:** This study aims to establish an effective nomogram to predict the overall survival of patients with intrahepatic cholangiocarcinoma (ICC).

**Patients and Methods:** Data used to build the nomogram comes from the Surveillance, Epidemiology, and End Results (SEER) database. Patients diagnosed with ICC between 2005 and 2016 were retrospectively collected. Prediction accuracy and discrimination ability of the nomogram was evaluated by concordance index (C-index) and calibration curve. The area under receiver operating characteristic (ROC) curve (AUC) and decision curve analysis (DCA) were used to compare the precision of the 1-, 3-, and 5-year survival of the nomogram with 8th American Joint Commission on Cancer (AJCC) tumor–node–metastasis (TNM) staging system. Finally, it was verified in a prospective study of patients diagnosed with ICC in the Second Affiliated Hospital of Nanchang University from 2013 to 2020 by bootstrap resampling.

**Result:** The study contains two parts of data; we establish a nomogram using external data, and we conducted internal verification and external verification. The nomogram that we have established has good calibration, with a concordance index (C-index) of 0.75 (95% CI, 0.74–0.76) for overall survival (OS) prediction. The AUC value of the nomogram predicting 1-, 3-, and 5-year OS rates were 0.821, 0.828, and 0.836, which were higher than those of the 8th AJCC TNM staging systems. The calibration curve for the probability of survival between prediction by nomogram and actual observation shows good agreement. The nomogram showed better accuracy than the 8th edition AJCC TNM staging.

**Conclusion:** The nomogram established can provide a more accurate prognosis for patients with intrahepatic cholangiocarcinoma.

## Introduction

Intrahepatic cholangiocarcinoma (ICC) originates from the epithelial cells of the intrahepatic bile duct, which can be a small intrahepatic bile duct or a large intrahepatic bile duct near the bifurcation of the hepatic duct (1). The incidence of ICC is second only to hepatocellular carcinoma (HCC) and accounts for ~5–30% of all liver malignancies (2, 3). In addition, the incidence and mortality of ICC have increased worldwide in recent years (4, 5). Unfortunately, the prognosis of patients with ICC, whether surgically or non-surgically, is not satisfactory (6, 7). ICC is significantly different from HCC in behavior, and the clinical features, imaging findings, and treatment methods of ICC are also different from HCC and distal cholangiocarcinoma (8). Therefore, ICC is a malignant tumor different from other tumors; it needs a unique prognostic prediction model of its own. A good predictive model can help doctors choose the best treatment to suit the individual prognosis of different patients. It is very important for our clinicians. At present, our most commonly used traditional staging system is the 8th edition American Joint Commission on Cancer (AJCC) system. A recent study (9) had proved that the AJCC system is not suitable for all ICC patients; it only considers tumor size, lymph node metastasis, and distant metastasis but does not consider other patient characteristics such as age, gender, and treatment methods. Therefore, we urgently need a staging system for the individual prognosis of ICC patients.

The purpose of this study is to develop a nomogram for predicting overall survival (OS) of ICC patients using a cohort from the Surveillance, Epidemiology, and End Results (SEER) database and to conduct internal and external verification. This nomogram can provide clinicians with a better tool for risk stratification, prognosis prediction, and therefore clinical decision.

## Patients and Methods

### Development Cohort

The SEER program of the National Cancer Institute provides data on cancer incidence and survival rates covering 30% of the US population. In this study, we collected patients diagnosed with ICC from 2005 to 2016 from SEER database, by using the SEER^*^Stat (National Cancer Institute, Bethesda, MD, USA) software version 8.3.8. The data we collected were from the International Classification of Diseases for Oncology 3rd edition (ICD-O-3), primary site code C22.1 (intrahepatic bile duct), along with histological/behavior code 8160.3 (cholangiocarcinoma). The exclusion criteria were as follows: (1) diagnosed under 18 years old, (2) combined with other primary tumors, (3) incomplete clinical data, (4) unclear follow-up information, and (5) surgical methods that did not achieve the purpose of treatment. Patients' clinical characteristics were extracted from the SEER database, including age at diagnosis, gender, tumor size, tumor–node–metastasis (TNM) stage, and follow-up information. The staging system uses the 8th AJCC edition system. The AJCC TNM 8th edition stage was calculated from the 6th or 7th edition TNM stages and other characteristics like tumor size (10, 11). OS refers to the time from diagnosis to death or the last follow-up. The approval and informed consent of the institutional review committee were exempted because the SEER database is a public database, which is open access for anyone who has registered an account and signed the authorization.

### External Validation Cohort

Eighty-eight patients diagnosed of ICC between 2013 and 2020 at the Second Hospital of Nanchang University were used as validation cohort. The criteria for the validation cohort and the inclusion criteria and exclusion criteria for the development cohort are fully chaired. The diagnosis of ICC patients who have not undergone surgery is based on clinical, radiographic, and serum markers (12). The clinical characteristics of all patients are collected from the electronic medical record. All procedures performed in this study involving human participants comply with the ethical standards of the institution and/or national research committee and with the 1964 Helsinki Declaration and amendment or similar ethical standards. External verification data have been approved by the Second Hospital of Nanchang University ethics committee.

### Statistical Analysis

Continuous data were presented by median ± range and compared with Student's *t*-test or Mann–Whitney *U*-test. Categorical data were presented by frequency (proportion) and compared with chi-square test. Cox regression model was used for multivariate analysis, and the independent risk factors that affect OS were extracted. The associated 95% confidence interval (CI) and hazard ratio (HR) were also calculated. OS were calculated by Kaplan–Meier curves and compared by the log-rank tests. Independent risk factors were used to establish a nomogram for 1-, 3-, and 5-year OS by using rms package in R Studio (3). The accuracy of the nomogram is evaluated by the C-index and the calibration curve (13).

The verification of the nomogram includes two parts: internal verification and external verification. First, we use the caret package of R Studio to divide the data collected from the SEER database into 30 and 70%, and the 30% of data were used for internal verification by bootstrap with 1,000 resamples. Second, clinical data from our institution were collected for external verification using the established nomogram. Lastly, the area under receiver operating characteristic (ROC) curve (AUC) was used to evaluate and compare the precision of the 1-, 3-, and 5-year survival of the nomogram. All statistical analyses were analyzed using SPSS version 26 (SPSS, Inc., Chicago, IL, USA) and R Studio version 1.3.1056 with R packages survival, rms, caret, survival ROC, and foreign packages. A two-tailed *P* < 0.05 was considered statistically significant.

## Result

### Patient Characteristics

A total of 1933 patients diagnosed with ICC were included from the SEER database during 2005 and 2016. Another 88 patients diagnosed with ICC were included as validation cohort from our institution during 2013 and 2020. The median age was 67 years (20–85 range) in the SEER database and 66 years (30–86 range) in the external validation cohort. The clinical characteristics are summarized in Table 1.

**Table 1:** Clinical characteristics of patients in the SEER database and external validation cohort.

**Characteristic**		**SEER database**		**Validation cohort**	
		**NO**	**%**	**NO**	**%**
Total		1,933	100	88	100
Gender	Female	950	49.1	47	53.4
	Male	983	50.9	41	46.6
Age	<50	196	10.1	7	8.0
	50–64	720	37.2	33	37.5
	65–79	792	41.0	39	44.3
	≥80	225	11.7	9	10.2
Surgery	No	1,347	69.7	31	35.2
	Yes	586	30.0	57	64.8
Radiotherapy	No	1,623	84.0	79	89.8
	Yes	310	16.0	9	10.2
Chemotherapy	No	874	45.2	39	44.3
	Yes	1,059	54.8	49	55.7
T stage (8th)	T1a	299	15.5	10	11.4
	T1b	348	18.0	25	28.4
	T2	883	45.7	33	37.5
	T3	278	14.3	15	17.0
	T4	125	6.5	5	5.7
LN metastasis	Absent	1,292	66.8	61	69.3
	Present	641	33.2	27	30.7
Metastasis	Absent	1,355	70.1	71	80.7
	Present	578	29.9	17	19.3
Tumor size	≤5 cm	708	36.6	39	44.3
	>5 cm	1,255	63.4	49	55.7

*LN, lymph node*.

### Os Analysis

**Figure 1:**
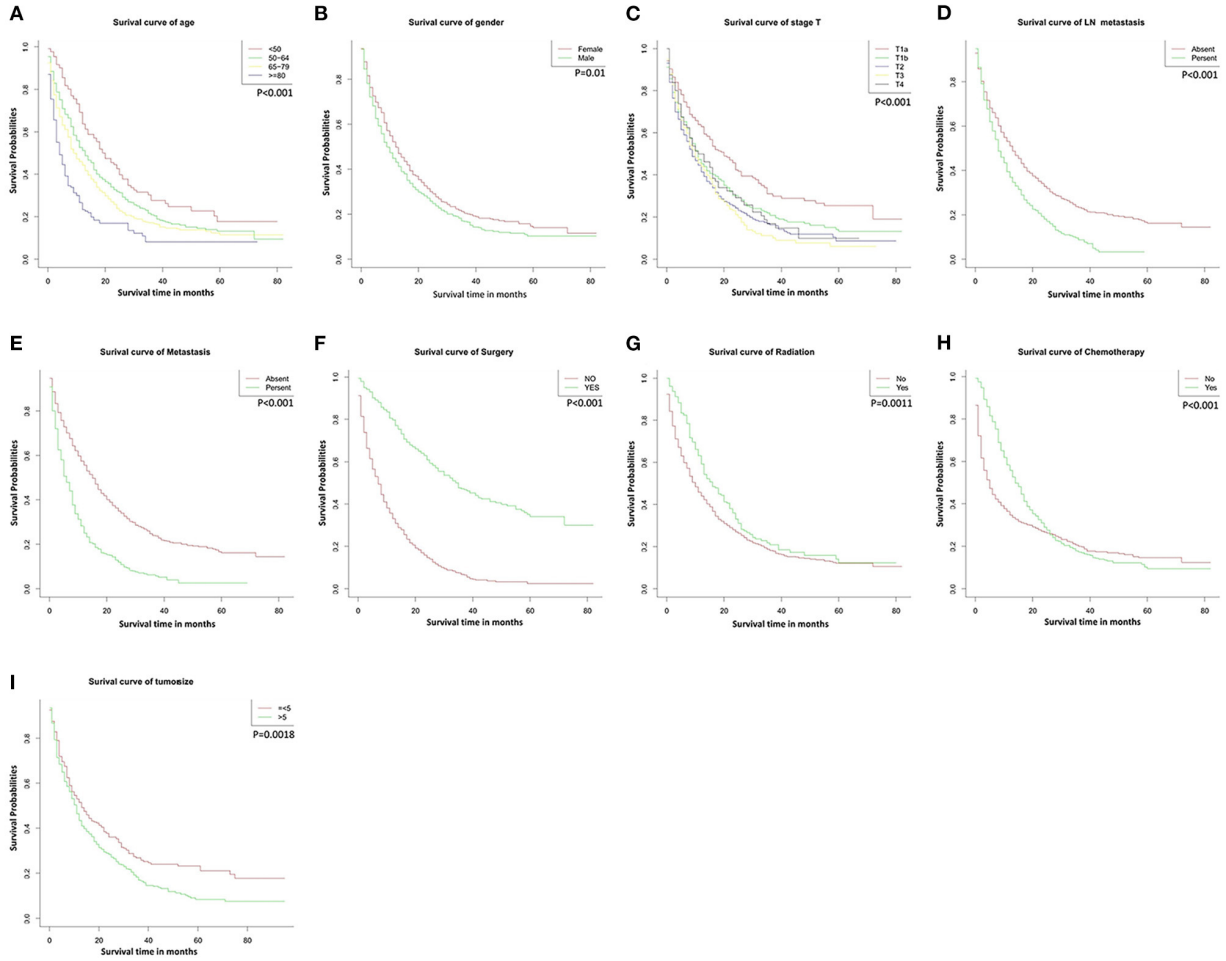
Overall survival rates according to patient characteristics: **(A)** Age; **(B)** Gender; **(C)** T stage; **(D)** LN metastasis; **(E)** Metastasis; **(F)** Surgery; **(G)** Radiation; **(H)** Chemotherapy; **(I)** Tumor size. LN, lymph node.

The median follow-up period was 11 months (0–83 range) in the development cohort and 14.5 months (1–79 range) in the validation cohort. The mortality rate was 77.9% (1505/1933), and the OS rates at 1, 3, and 5 years were 44.9, 9.1, and 3.1%. Kaplan–Meier curve analysis showed significant differences according to different characteristics. No surgical resection (*P* < 0.001, Figure 1F), tumor size >5 cm (P < 0.05, Figure 1I), older age (*P* < 0.001, Figure 1A), lymph node metastases (*P* < 0.001, Figure 1D), distant metastases (*P* < 0.001, Figure 1E), higher T stage (*P* < 0.001, Figure 1C), no radiotherapy (*P* < 0.001, Figure 1G), no chemotherapy (*P* < 0.001, Figure 1H), and male gender (*P* < 0.05, Figure 1B) showed poorer OS.

### Univariate and Multivariate Analyses of Effects of Factors on OS

In the COX regression univariate analysis, age, gender, surgical treatment, radiation treatment, chemotherapy, T stage, N stage, M stage, and tumor size were significantly correlated to the OS of ICC patients. After adjusting for other risk factors, in the multivariate COX regression analysis, age, surgical treatment, T stage, N stage, M stage, radiation treatment, and chemotherapy were significantly correlated to the OS of ICC patients (Table 2).

**Table 2:** Univariate and multivariate analyses for OS in patients with ICC.

**Characteristic**		**Univariate analysis**			**Multivariate analysis**		
		**HR**	**95% CI**	***P***	**HR**	**95% CI**	***P***
Gender	Female	Ref			Ref		
	Male	1.176	1.040–1.329	0.009	1.088	0.961–1.231	0.18
Age	<50	Ref			Ref		
	50–64	1.380	1.096–1.736	0.006	1.159	0.919–1.463	0.21
	65–79	1.647	1.312–2.068	<0.001	1.460	1.158–1.841	<0.001
	≥80	2.588	1.978–3.387	<0.001	1.634	1.229–2.173	<0.001
Surgery	No	Ref			Ref		
	Yes	0.253	0.215–0.299	<0.001	0.239	0.200–0.287	<0.001
Radiotherapy	No	Ref			Ref		
	Yes	0.750	0.630–0.891	0.001	0.688	0.576–0.821	<0.001
Chemotherapy	No	Ref			Ref		
	Yes	0.712	0.629–0.805	<0.001	0.405	0.350–0.469	<0.001
T stage (8th)	T1a	Ref			Ref		
	T1b	1.452	1.164–1.811	<0.001	1.180	0.896–1.553	0.24
	T2	1.728	1.423–2.098	<0.001	1.491	1.188–1.872	<0.001
	T3	1.790	1.422–2.252	<0.001	1.541	1.186–2.004	0.001
	T4	1.513	1.124–2.037	0.006	1.503	1.094–2.065	0.01
LN metastasis	Absent	Ref			Ref		
	Present	1.504	1.323–1.710	<0.001	1.297	1.127–1.492	<0.001
Metastasis	Absent	Ref			Ref		
	Present	2.110	1.852–2.403	<0.001	1.589	1.371–1.841	<0.001
Tumor size	≤5 cm	Ref			Ref		
	>5 cm	1.292	1.135–1.471	<0.001	1.050	0.891–1.238	0.56

*Cox regression analyses. OS, overall survival; ICC, intrahepatic cholangiocarcinoma; HR, hazard ratio; CI, confidence interval; LN, lymph node*.

### Nomogram Construction and Validation for OS

**Figure 2:**
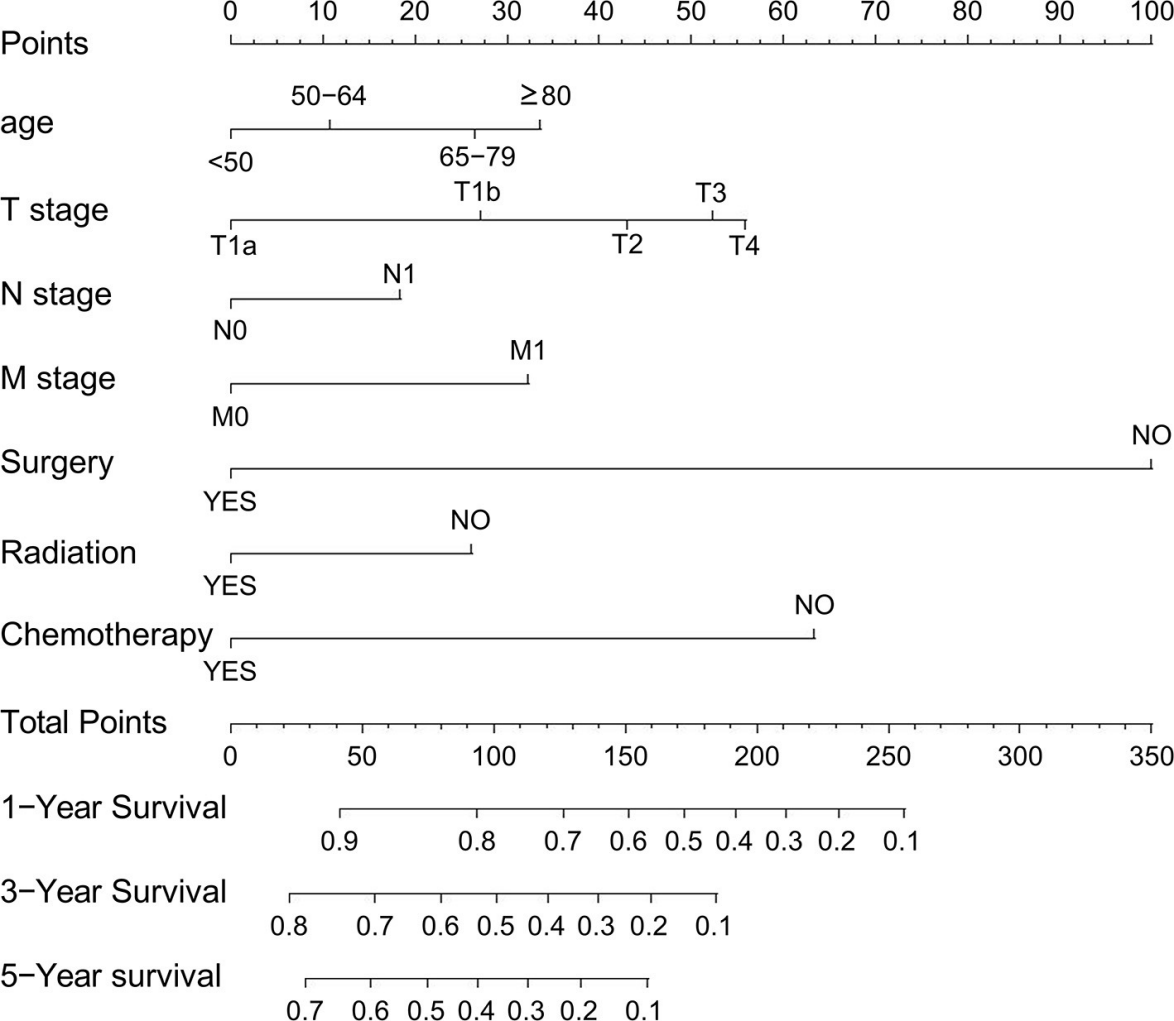
Nomogram predicting 1-, 3-, and 5-year OS of patients with ICC. OS, overall survival.

**Figure 3:**
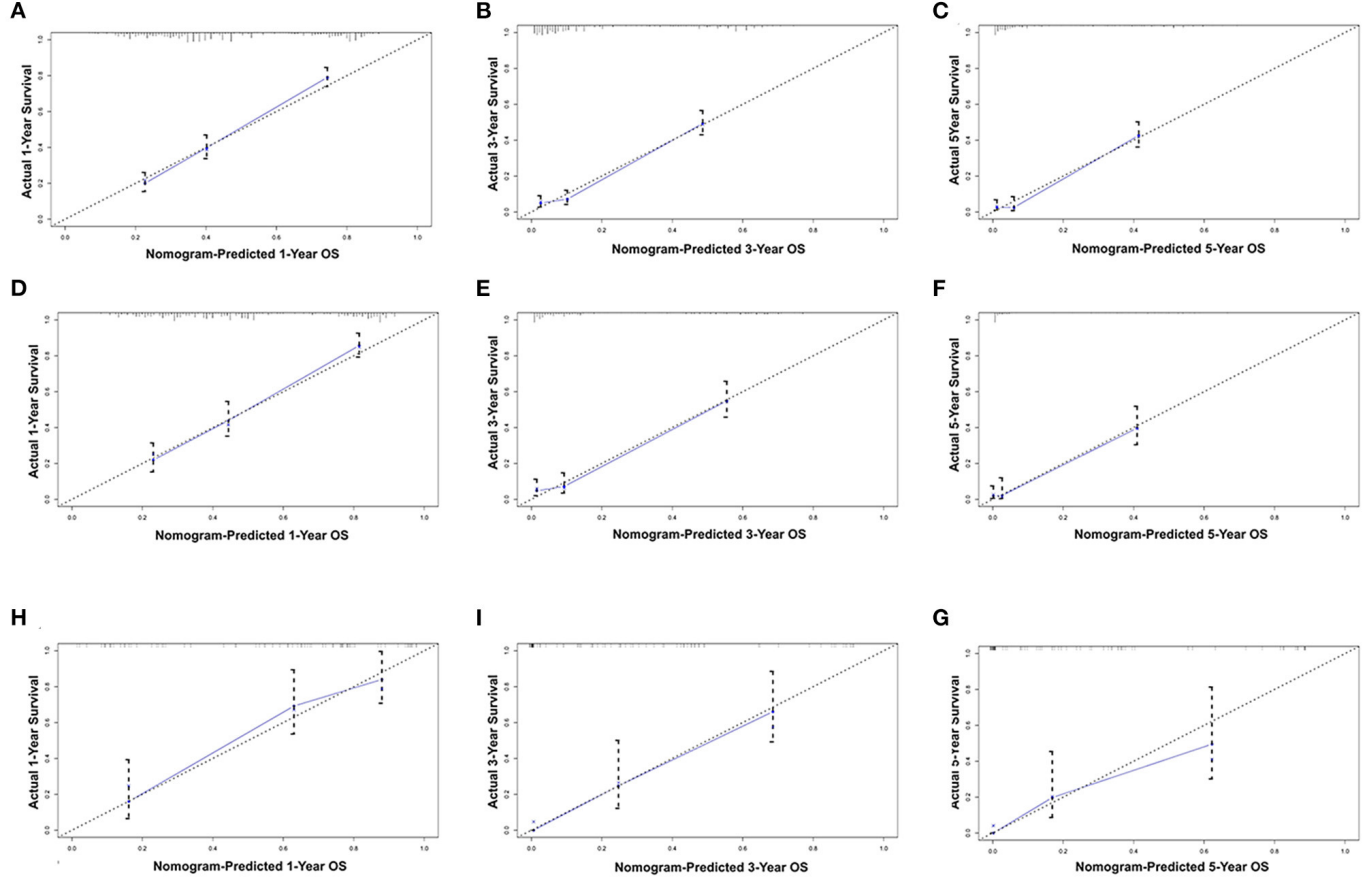
Calibration plots of the nomogram for 1-, and 3-year OS prediction of the training set **(A–C)** and internal verification set **(D–F)** and external verification set **(G–I)**. X-axis represents the nomogram-predicted probability of survival; Y-axis represents the actual OS probability. A perfectly accurate nomogram prediction model would result in a plot that the observed and predicted probabilities for given groups fall along the line. Dots with bars represent nomogram-predicted probabilities along with 95% confidence interval. OS, overall survival.

All the independent risk factors that have a significant impact on OS were included in the nomogram for predicting 1-, 3-, and 5-year OS in the training set (Figure 2). By adding the variable scores corresponding to each patient, it is easy to get the survival probability of different individuals. The C-index shown in the nomogram was 0.754 (95% CI, 0.746–0.762), with good accuracy. During internal verification, the nomogram showed good accuracy with C-index of 0.761 (95% CI, 0.751–0.771). In external verification, the nomogram also showed good accuracy with a C-index of 0.767 (95% CI, 0.735–0.799). In the 8th AJCC TNM staging system, the C-index is 0.607 (95% CI, 0.598–0.612). In the internal and external verifications, the 1-, 3-, and 5-year calibration curves showed that the survival rates predicted by the nomogram were in good agreement with the actual survival rates (Figure 3).

### Survival Analysis According to the Risk Stratification Based on the Nomogram

**Figure 4:**
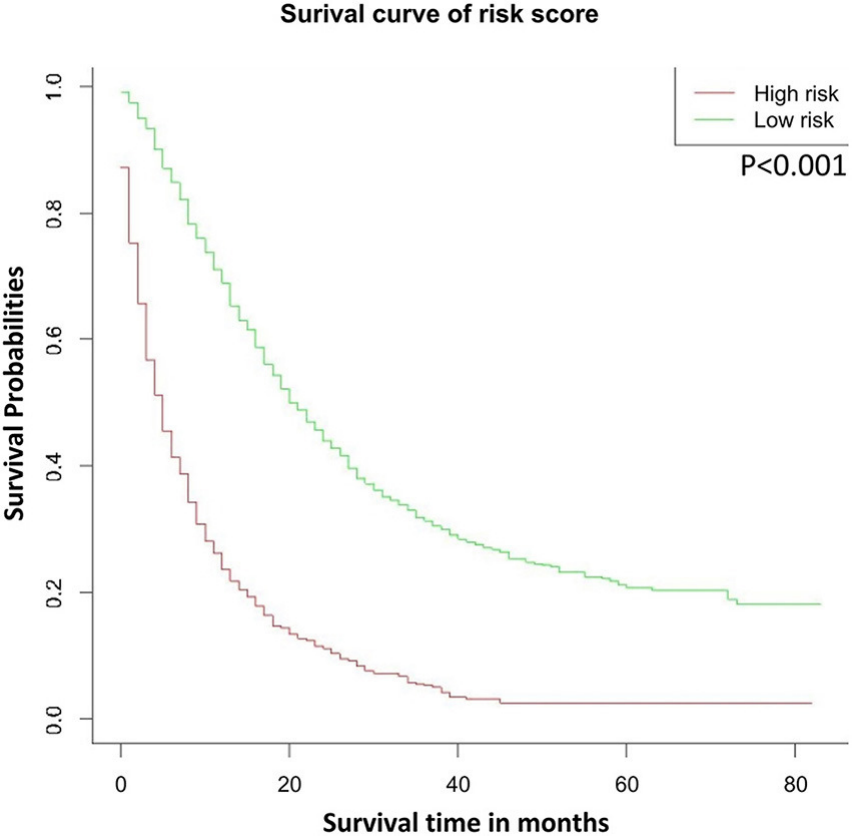
OS stratified by the risk levels of the nomogram-predicted survival probabilities. OS, overall survival.

We divided the probability scores of all patients into two parts according to the average number. Patients with higher scores than the average are defined as high risk, and those lower are defined as low risk. As shown in Figure 4, we could see that the survival rate of low-risk patients was significantly higher than that of high-risk patients (*P* < 0.001).

### Comparison of the Performance of the Nomogram and 8th AJCC TNM Stage System

**Figure 5:**
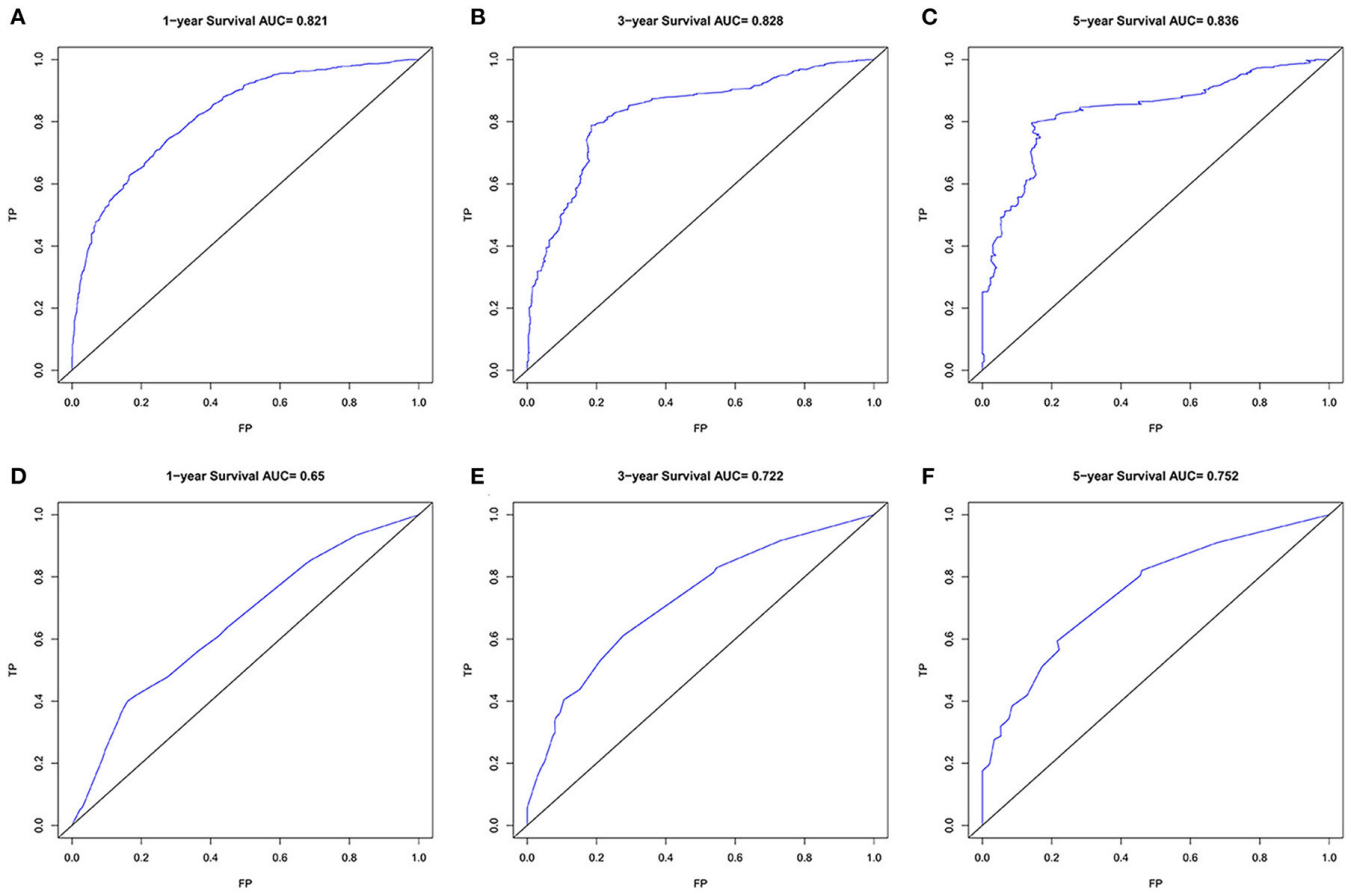
The ROC curves of the nomogram **(A–C)** and the 8th AJCC TNM staging system **(D–F)** for 1-, 3-, and 5-year OS prediction. OS, overall survival.

**Figure 6:**
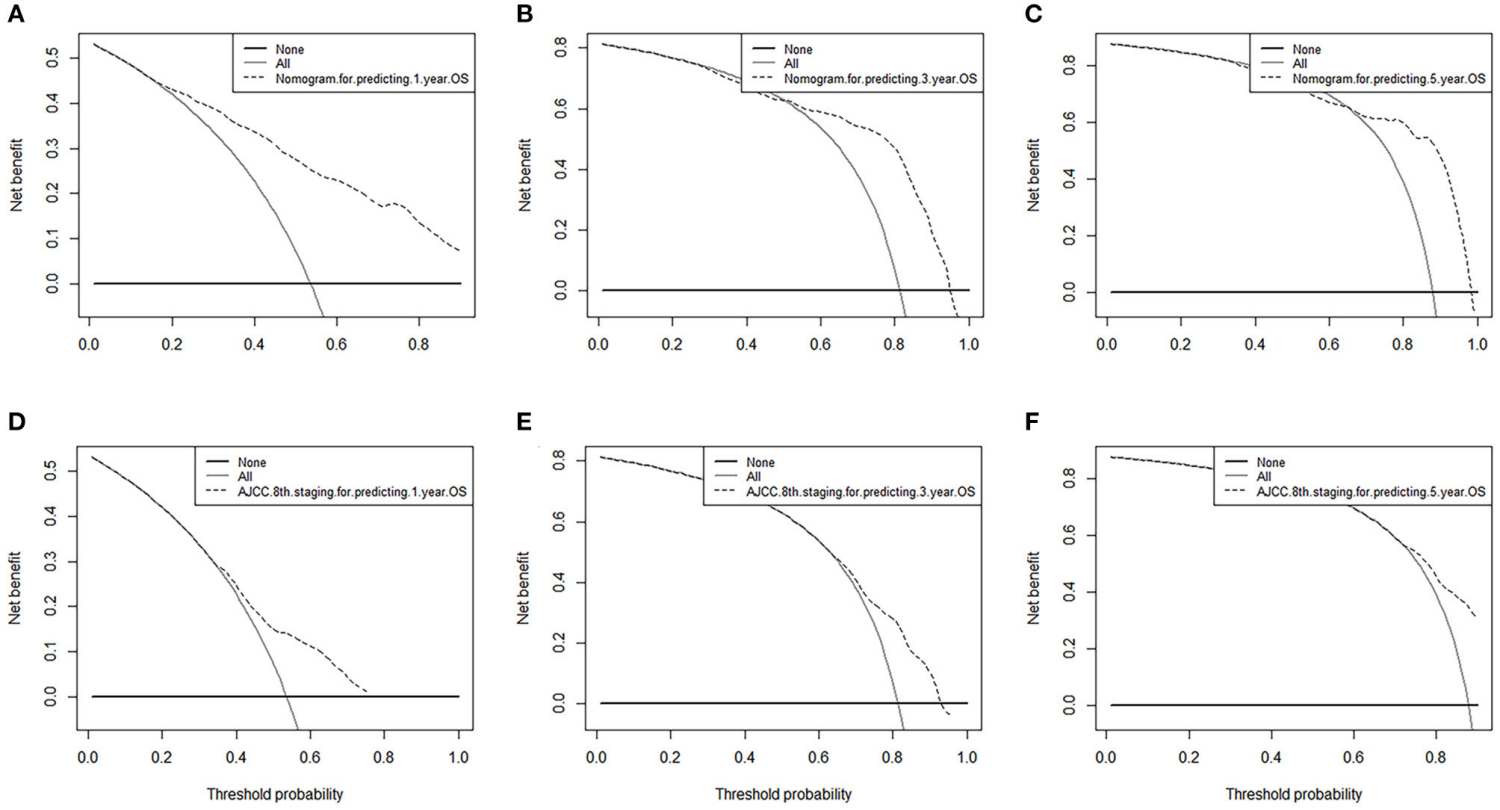
Decision curve analysis of nomograms and AJCC 8th edition staging system for predicting **(A,D)** 1-year OS; **(B,E)** 3-year OS; **(C,F)** 5-year OS.

Finally, we analyzed the value of AUC to compare the discriminative ability of the established nomogram and the 8th edition TNM staging system (Figure 5). For the entire development cohort, the AUC values of the nomogram used to predict 1-, 3-, and 5-year OS were, respectively, 0.821, 0.828, and 0.836. However, the AUC values of the 8th AJCC TNM staging system were 0.650, 0.722, and 0.752. In DCA, within a wide range of threshold probabilities, the established nomogram had a higher net benefit in predicting 1-, 3-, and 5-year OS compared with the 8th AJCC TNM staging system (Figure 6). In general, the nomogram we have established had a better recognition ability and precision than the 8th AJCC TNM staging system.

## Discussion

The current study used data from the SEER database to establish a nomogram for predicting patients diagnosed with ICC and verified both internally and externally. The internal verification data also came from the SEER database, while the external verification data came from an independent cohort collected by our hospital. The nomogram showed a good distinction and calibration function, which provided better clinical decision making for both patents and clinicians.

ICC is the second most common liver malignant tumor following hepatocellular carcinoma (14), and the incidence of ICC is increasing worldwide (15). It is significant for clinicians to make individualized prognostic judgments based on accurate patient information. Traditional staging system such as the 8th AJCC TNM staging system only included specific related variables and evaluated the prognosis of specific patients. A recent study has shown that the TNM staging of the 8th edition of AJCC had a moderate discrimination ability in predicting the OS of ICC patients, while there was no significant improvement in the overall prognosis compared with the 7th edition (16). Therefore, it is meaningful to establish a novel prediction system that can effectively predict the prognosis of ICC patients. Recent studies paid more attention to ICC patients who underwent surgery (2, 17, 18). The C-index of the nomogram established by us is higher than that of the 8th edition AJCC TNM staging and had better predictive ability.

Although the prognosis of ICC patients who have undergone surgery was much better than that of those who have not undergone surgery, most patients have lost the chance of radical surgery due to locally advanced or distant metastases at the time of diagnosis (19–21). In our research, there is a fact that most patients have not undergone surgery; therefore, it is very necessary to include patients who have not undergone surgery into the study. Moreover, in our research, it could be found that some advanced patients who have not received surgery have a considerable prognosis; this is not seen in other similar studies. Many recent studies have shown that tumor-related factors, such as tumor size, tumor invasion, and lymph node condition, have a certain impact on the prognosis of ICC patients (22–24). The AJCC system is developed based on these related factors, and the latest system has been updated to the eighth edition, with using TNM staging to represent the degree of tumor invasion, lymph node metastasis, and a distant metastasis. Radiotherapy and chemotherapy now play a very important role in tumor treatment. In our study, radiotherapy and chemotherapy showed a good correlation in the survival rate of patients.

In our study, we can see that higher-level TNM staging has a poor prognosis of OS. In addition, multivariate analysis showed that TNM stages are independent risk factors that affect the prognosis of ICC patients. As previously reported (25), lymph node metastasis will have an impact on the patient's postoperative review, so the lymph node affects the patient's prognosis to a certain extent.

In our time, for clinicians, individualized cancer treatment is particularly important, and on this basis, we established a nomogram. Our nomogram combines factors that are easily obtained from the clinic, which makes it easy to calculate the individualization of ICC patients. In our research, whether it is the nomogram, internal verification, or external verification, there is a relatively good C-index and calibration curve, and we compared it with the eighth edition of TNM staging with a higher C-index. The larger the C-index, to a certain extent, the more accurate the prognosis prediction (26). However, high prognostic prediction accuracy does not necessarily have good clinical applicability (27). Decision curve analysis is a novel way to evaluate models; it uses an estimated threshold probability distribution, and the weighted area under the net income curve is used as a summary metric to compare risk prediction models in the range of interest (28–30). Therefore, we quoted DCA to evaluate our nomogram and compared it with the 8th TNM staging system. The results show that our nomogram has better clinical applicability.

This study has several limitations. First, we cannot find in the SEER database the serological tests that may have an impact on OS in ICC patients, such as tumor markers and blood routines, and some related positive variables, such as surgical margins and vascular invasion, cannot be found either. These variables may be a supplement to our current stage, which will be the main part of our future research. Second, like other retrospective studies, both development and validation cohorts are affected by selection bias. Last, due to the lack of external verification data, factors that can be found in the SEER database cannot be included in the study, and the small amount of external verification samples by a single institution may lead to verification errors. More samples and multi-institution verification will be conducted to verify the accuracy of the nomogram. However, despite these limitations, we have established a nomogram with better clinical applicability and better than 8th TNM staging system.

## Conclusion

All in all, we build a nomogram to predict 1-, 3-, and 5-year diagnosis ICC based on a large population. This nomogram integrates easily accessible factors and has been verified internally and externally, showing good accuracy and clinical applicability, which may help clinicians to implement individualized clinical decisions.

## Data Availability Statement

The raw data supporting the conclusions of this article will be made available by the authors, without undue reservation.

## Ethics Statement

Ethical review and approval was not required for the study on human participants in accordance with the local legislation and institutional requirements. Written informed consent for participation was not required for this study in accordance with the national legislation and the institutional requirements.

## Author Contributions

SZ was responsible for conception, design, quality control of this study, reviewed, and edited the manuscript. CY, ZH, SZ, and KW performed the study selection, data extraction, statistical analyses, and were major contributors in writing the manuscript and contributed in classification criteria discussion. CY and ZH participated in studies selection and statistical analyses. All authors have read and approved the final version of the manuscript.

## Conflict of Interest

The authors declare that the research was conducted in the absence of any commercial or financial relationships that could be construed as a potential conflict of interest.
